# Development and validation of the first species-specific MALDI-TOF MS main spectra profile database for rapid identification of Aeromonas schubertii associated with white nodular disease in freshwater fish

**DOI:** 10.14202/vetworld.2026.4175-4190

**Published:** 2026-09-22

**Authors:** Muhammad Fadhlullah Mursalim, Hendri Budiyansah, Mahmoud Mabrok, Sulaiman F. Aljasir, Rian Hari Suharto, Channarong Rodkhum

**Affiliations:** 1 Veterinary Paramedic Study Program, Faculty of Vocational Studies, Hasanuddin University, Makassar 90245, South Sulawesi, Indonesia.; 2 Veterinary Study Program, Faculty of Medicine, Hasanuddin University, Makassar 90245, South Sulawesi, Indonesia.; 3 The International Graduate Course of Veterinary Science and Technology, Faculty of Veterinary Science, Chulalongkorn University, Bangkok, Thailand.; 4 Department of Aquatic Animal Medicine, Faculty of Veterinary Medicine, Suez Canal University, Ismailia 41522, Egypt.; 5 Department of Veterinary Preventive Medicine, College of Veterinary Medicine, Qassim University, Buraydah 52571, Saudi Arabia.; 6 Center of Excellence in Fish Infectious Diseases Research Unit, Faculty of Veterinary Science, Chulalongkorn University, Bangkok, Thailand.; 7 Department of Veterinary Microbiology, Faculty of Veterinary Science, Chulalongkorn University, Bangkok, Thailand.

**Keywords:** Aeromonas schubertii, aquaculture diagnostics, bacterial identification, freshwater fish, MALDI-TOF MS, main spectra profile, spectral database, white nodular disease

## Abstract

**Background and Aim:**

*Aeromonas schubertii* is an emerging aquatic bacterial pathogen associated with distinctive white nodular disease in freshwater fish and is increasingly relevant to aquaculture health and disease surveillance. Accurate species-level identification remains challenging because commercial matrix-assisted laser desorption/ionization time-of-flight mass spectrometry (MALDI-TOF MS) databases have limited species-specific spectral representation of *A. schubertii*. This study aimed to develop and validate the first species-specific main spectra profile (MSP) database for rapid and reliable identification of *A. schubertii*.

**Materials and Methods:**

Six representative *A. schubertii* isolates were used to construct MSPs following ethanol–formic acid–acetonitrile protein extraction. A minimum of 20 high-quality spectra per isolate were retained for MSP generation. The customized database was evaluated using 19 *A. schubertii* isolates, including the six isolates used for database construction and 13 additional isolates. Identification reliability and repeatability were evaluated, and discriminatory performance was assessed against four closely related non-target *Aeromonas* species. Principal component analysis (PCA), spectral profiling, and dendrogram analyses were used to evaluate spectral relatedness and species differentiation.

**Results:**

Using the commercial database alone, 15/19 (78.95%) isolates achieved reliable species-level identification (log scores of 2.01–2.22), whereas 4/19 (21.05%) were identified only at the genus level (scores of 1.95–1.98). After incorporating the customized MSP database, all 19 isolates (100%) were correctly identified as *A. schubertii*, with highly reliable log scores ranging from 2.49 to 2.68. Technical replicates showed high spectral reproducibility, with Pearson correlation coefficients of 0.95–0.97. PCA and dendrogram analyses clearly separated *A. schubertii* from the tested non-target *Aeromonas* species, supporting the discriminatory performance of the customized database. Molecular analysis of 16S rRNA and *gyrB* sequences further supported the taxonomic identification of the isolates.

**Conclusion:**

The species-specific MSP database substantially improved MALDI-TOF MS identification of *A. schubertii*, increasing species-level identification from 78.95% to 100%. The database provides a rapid and reliable framework for improving diagnosis and surveillance of this emerging aquaculture pathogen. Further expansion with geographically diverse isolates and multicenter validation is warranted to strengthen database transferability and broader diagnostic application.

## INTRODUCTION

*Aeromonas schubertii* is a motile, rod-shaped, Gram-negative bacterium characterized by a single polar flagellum and taxonomically assigned to the family Aeromonadaceae. Members of the genus *Aeromonas* are widely distributed across diverse ecological niches, including soil, freshwater and marine environments, and the intestinal tracts of animals, reflecting their considerable environmental adaptability [1, 2]. *A. schubertii* has been recognized as an opportunistic pathogen of relevance to human health and has been associated with acute gastroenteritis, with environmental exposure and direct contact representing potential transmission routes [3]. Beyond its public health relevance, *A. schubertii* has emerged as an important pathogen in aquaculture. Infections have been documented in several economically important aquatic species, including the marine shrimp *Penaeus monodon* and *Litopenaeus vannamei* [3, 4], mussels [5], and Nile tilapia (*Oreochromis niloticus*) [6]. More recently, *A. schubertii* has been implicated in severe disease outbreaks in snakehead fish, including *Channa argus*, *Channa striata*, and *Channa maculata*, with reported mortality reaching 45% over 40 days [2, 7, 8]. Such outbreaks can cause substantial economic losses through reduced fish survival, increased treatment costs, and decreased farm productivity.

Notably, *A. schubertii*-associated outbreaks in freshwater fish are characterized by distinctive white nodular lesions distributed across internal organs, an atypical pathological manifestation that is infrequently reported in other bacterial infections of fish [7, 9]. The combination of substantial mortality and characteristic pathological changes highlights the importance of timely and accurate identification of *A. schubertii* for disease diagnosis, epidemiological surveillance, and outbreak management in aquaculture systems.

Accurate species-level identification of *Aeromonas* remains a persistent challenge in diagnostic microbiology. Conventional biochemical methods are time-consuming and may produce ambiguous or erroneous results because of phenotypic variability and overlapping characteristics among closely related species [10]. Molecular approaches and whole-genome sequencing provide greater taxonomic resolution; however, their routine application may be constrained by methodological complexity, cost, and reported inconsistencies in species discrimination [11–13]. These limitations can delay reliable species-level identification and complicate epidemiological investigations and disease-management decisions. Routine diagnostic workflows for *A. schubertii* commonly integrate classical bacteriological techniques with polymerase chain reaction (PCR)-based molecular assays [14–16]. These approaches may involve multiple sequential steps, including bacterial isolation, cultivation, physiological and biochemical characterization, and confirmatory molecular testing, resulting in labor-intensive, time-consuming workflows. Therefore, rapid, reliable, and high-throughput species-level identification remains important for strengthening diagnostic capacity, particularly in Southeast Asian aquaculture settings.

Matrix-assisted laser desorption/ionization time-of-flight mass spectrometry (MALDI-TOF MS) has emerged over the past two decades as a powerful approach for microbial identification in clinical and research laboratories [17–19]. MALDI-TOF MS offers several practical advantages, including rapid analysis, relatively low per-sample operating costs, high-throughput capacity, and the ability to generate identification results within minutes after colony isolation. However, its identification accuracy depends strongly on the quality, diversity, and completeness of the reference spectral database [20]. Consequently, microorganisms absent or poorly represented in commercial databases may receive low-confidence scores, be identified only at the genus level, or be misclassified.

Despite the increasing application of MALDI-TOF MS in aquatic animal diagnostics, reliable species-level identification of *A. schubertii* remains constrained by its limited species-specific spectral representation in existing reference databases. This limitation is particularly important for isolates associated with white nodular disease in freshwater fish, for which rapid differentiation from closely related *Aeromonas* species is required for accurate diagnosis and epidemiological surveillance. Although previous work has investigated MALDI-TOF MS-based differentiation of multiple fish-associated *Aeromonas* species, a dedicated species-specific main spectra profile (MSP) database focused on *A. schubertii* has not been established. To our knowledge, no previous study has developed and validated a dedicated MSP database specifically for *A. schubertii*, particularly using isolates associated with white nodular disease in freshwater fish. Therefore, the lack of a curated species-specific spectral reference library represents an important diagnostic and methodological gap that may limit the accuracy and confidence of MALDI-TOF MS-based identification of this emerging aquatic pathogen.

To address this diagnostic gap, the present study aimed to develop and validate a species-specific MSP database for *A. schubertii* using the MALDI Biotyper Compass platform (Bruker Daltonics). Specifically, the study sought to generate high-quality and reproducible MSPs from representative *A. schubertii* isolates, assess whether incorporation of the customized MSP database improved species-level identification compared with the existing commercial reference database, evaluate identification repeatability, and determine the discriminatory capacity of the developed database against closely related non-target *Aeromonas* species using spectral and multivariate analyses. By focusing on *A. schubertii*, the study was designed to provide a practical framework for improving rapid, reliable identification of this underrepresented pathogen and to support future diagnostic surveillance of *A. schubertii*-associated disease in freshwater aquaculture.

## MATERIALS AND METHODS

### Ethical approval

All procedures involving bacterial handling, culture, and MALDI-TOF MS sample preparation were conducted in a Biosafety Level 2 (BSL-2) laboratory in accordance with the institutional biosafety requirements of Chulalongkorn University. The laboratory procedures were reviewed and approved by the Institutional Biosafety Committee of Chulalongkorn University (Approval No. 2031043). Moribund fish used for diagnostic sampling were euthanized at the sampling sites by immersion in clove oil (1 g/L) for approximately 10 min in accordance with the previously described procedure [20]. Internal organs were subsequently collected aseptically for bacteriological examination.

### Study period and location

The study was conducted over a three-month period, from August to November 2025. The bacterial isolates investigated in this study originated from diseased freshwater fish sampled from July 2019 to July 2020 in Thailand and Vietnam. Thai isolates were obtained from Samut Sakhon and Nakhon Pathom Provinces, whereas Vietnamese isolates originated from Tra Cu District, Tra Vinh Province; Chau Phu District, An Giang Province; and Thanh Binh District, Dong Thap Province. The isolates were submitted to the Center of Excellence in Fish Infectious Diseases, Faculty of Veterinary Science, Chulalongkorn University, Thailand, for diagnostic examination and further characterization. The six isolates selected for MSP database construction were recovered from diseased snakehead fish collected in Nakhon Pathom Province, Thailand, in 2020.

### Study design

This laboratory-based diagnostic method development and validation study aimed to construct and evaluate a species-specific MALDI-TOF MS MSP database for *A. schubertii*. Among 81 bacterial isolates obtained from diseased freshwater fish, 19 were identified as *A. schubertii* and included in the study. Six representative isolates (CUVETASC03–CUVETASC08) were used for MSP library construction, whereas the complete set of 19 isolates, comprising the six MSP development isolates and 13 additional isolates, was used to assess identification performance. Four closely related non-target *Aeromonas* species were included as outgroup controls for assessment of spectral discrimination and clustering. Molecular confirmation of the six MSP development isolates was performed using *16S rRNA* and *gyrB* sequence analyses.

### Bacterial isolation and identification

Among 81 bacterial isolates obtained from diseased freshwater fish during 2019–2020, 19 were identified as *A. schubertii* (Table 1). Of these, 17/19 isolates were recovered from striped snakehead (*Channa striata*), one from Asian seabass (*Lates calcarifer*), and one from hybrid red tilapia (*Oreochromis* sp.).

Moribund fish were euthanized at the sampling sites by immersion in clove oil (1 g/L) for approximately 10 min, as previously described [20]. Samples from internal organs, including the liver, spleen, and kidney, were collected aseptically using sterile inoculating loops and streaked onto tryptic soy agar (TSA; Bacto™, Becton, Dickinson and Company [BD], Sparks, MD, USA) supplemented with 5% sheep blood. The inoculated plates were incubated at 28°C for 24 h. Representative colonies with distinct morphologies were selected for preliminary identification using conventional microbiological procedures, including Gram staining and catalase and oxidase tests. The resulting phenotypic characteristics were consistent with classification within the genus *Aeromonas*. Confirmed isolates were preserved in tryptic soy broth (TSB; Bacto™) supplemented with 20% glycerol and stored at −80°C until further analysis.

### PCR assay, DNA sequencing, and phylogenetic analysis based on *16S rRNA* and *gyrB* sequences

Species-level identification of the six isolates selected for MSP development was confirmed by PCR amplification and sequencing of the *16S rRNA* and *gyrB* genes. The *16S rRNA* gene was amplified using primers 27F (5′-AGAGTTGATCATGGCTCAG-3′) and 1492R (5′-GGTTCACTTGTTACGACTT-3′) [21], whereas *gyrB* was amplified using primers gyrB3-F (5′-TCCGGCGGTCTGCACGGCGT-3′) and gyrB14-R (5′-TTGTCCGGGTTGTACTCGTC-3′) [9, 22]. The expected amplicon sizes were approximately 1500 bp for the *16S rRNA* gene and 1100 bp for *gyrB*.

**Table 1:** Summary of A. schubertii isolates investigated in this study.

**Isolate code**	**Host/source**	**Sampling location**	**Country**	**Year**	***16S rRNA*** **accession No.**	***gyrB*** **accession No.**
CUVETASC01	Seabass	Samut Sakhon Province	Thailand	2019	ND	ND
CUVETASC02	Hybrid red tilapia	Samut Sakhon Province	Thailand	2019	ND	ND
CUVETASC03	Snakehead	Nakhon Pathom Province	Thailand	2020	ON007120	ON381189
CUVETASC04	Snakehead	Nakhon Pathom Province	Thailand	2020	ON624238	ON409527
CUVETASC05	Snakehead	Nakhon Pathom Province	Thailand	2020	ON651659	ON660882
CUVETASC06	Snakehead	Nakhon Pathom Province	Thailand	2020	ON651660	ON660883
CUVETASC07	Snakehead	Nakhon Pathom Province	Thailand	2020	ON820210	ON660884
CUVETASC08	Snakehead	Nakhon Pathom Province	Thailand	2020	ON820211	ON660885
CUVETASC09	Snakehead	Nakhon Pathom Province	Thailand	2020	ND	ND
CUVETASC10	Snakehead	Nakhon Pathom Province	Thailand	2020	ND	ND
CUVETASC11	Snakehead	Nakhon Pathom Province	Thailand	2020	ND	ND
CUVETASC12	Snakehead	Nakhon Pathom Province	Thailand	2020	ND	ND
CUVETASC13	Snakehead	Tra Cu District, Tra Vinh Province	Vietnam	2019	ND	ND
CUVETASC14	Snakehead	Tra Cu District, Tra Vinh Province	Vietnam	2019	ND	ND
CUVETASC15	Snakehead	Chau Phu District, An Giang Province	Vietnam	2019	ND	ND
CUVETASC16	Snakehead	Chau Phu District, An Giang Province	Vietnam	2019	ND	ND
CUVETASC17	Snakehead	Thanh Binh District, Dong Thap Province	Vietnam	2019	ND	ND
CUVETASC18	Snakehead	Thanh Binh District, Dong Thap Province	Vietnam	2019	ND	ND
CUVETASC19	Snakehead	Thanh Binh District, Dong Thap Province	Vietnam	2019	ND	ND

ND = not deposited in GenBank.

Genomic DNA was extracted from the tested isolates using the boiling method [23]. Amplification of the *16S rRNA* gene was performed with an initial denaturation at 94°C for 5 min, followed by 35 cycles of denaturation at 94°C for 1 min, annealing at 56°C for 30 s, and extension at 72°C for 90 s, followed by a final extension at 72°C for 10 min. Amplification of *gyrB* was performed with an initial denaturation at 94°C for 2 min, followed by 30 cycles of denaturation at 94°C for 30 s, annealing at 56°C for 30 s, and extension at 72°C for 2 min, followed by a final extension at 72°C for 5 min.

PCR products were visualized on 1.5% agarose gels stained with RedSafe™ and purified using the NucleoSpin® Gel and PCR Clean-up Kit (Macherey-Nagel, Germany). Purified amplicons were subjected to Sanger sequencing, and forward and reverse sequences were assembled using BioEdit version 7.1.1. Species identification was confirmed using Basic Local Alignment Search Tool (BLAST) against the National Center for Biotechnology Information (NCBI) database, applying sequence-identity thresholds of ≥99.5% for *16S rRNA* and ≥99.0% for *gyrB*. Sequences were aligned using ClustalW, and maximum-likelihood phylogenetic trees were constructed using MEGA X version 10.2.6 with the Tamura–Nei model and 1,000 bootstrap replicates. Gaps and missing data were handled using the partial-deletion option with a site-coverage cutoff of 95% [24–26].

### Preparation of samples for MALDI-TOF MS

All *A. schubertii* isolates were recovered from frozen stocks and subcultured on TSA (Bacto™) at 28°C for 24 h. Well-isolated colonies were subjected to protein extraction using the standard ethanol–formic acid–acetonitrile protocol for MALDI-TOF MS analysis. Briefly, 1 µL of extracted protein was spotted onto a 96-position polished-steel MALDI target plate, overlaid with α-cyano-4-hydroxycinnamic acid (HCCA) matrix, and air-dried at room temperature. Samples were analyzed using a microflex® LRF MALDI-TOF MS instrument (Bruker Daltonics, Bremen, Germany), and the resulting spectra were compared with the MALDI Biotyper® (Bruker Daltonics) reference library [27].

### MALDI-TOF MS extraction and database construction

Six representative *A. schubertii* isolates (CUVETASC03–CUVETASC08) were selected for MSP library cons-truction to incorporate multiple independent isolates and capture intraspecies spectral variability. These isolates originated from diseased snakehead fish collected in Nakhon Pathom Province, Thailand, in 2020. The isolates were recovered from glycerol stocks, subcultured on TSA, and subjected to ethanol–formic acid–acetonitrile protein extraction.

Briefly, bacterial biomass was suspended in 300 µL of high-performance liquid chromatography-grade water and 900 µL of absolute ethanol, centrifuged, air-dried, and subsequently extracted with 30 µL of formic acid and 30 µL of acetonitrile. A 1 µL aliquot of the resulting supernatant was spotted onto a polished-steel MALDI target plate and overlaid with HCCA matrix. Following mass calibration, smoothing, normalization, baseline correction, peak detection, and outlier removal, spectra were analyzed using FlexAnalysis software version 3.0 (Bruker Daltonics) in positive linear mode over a mass range of 2–20 kDa.

For MSP generation, each isolate was analyzed using eight independent spots, with three spectra acquired from each spot, yielding 24 raw spectra per isolate. Spectra showing weak signal intensity, irregular baselines, peak shifting, or poor reproducibility were excluded. A minimum of 20 high-quality spectra per isolate were retained for MSP construction using MBT Compass Explorer® (Bruker Daltonics) [28, 29]. MSPs were accepted when reproducible peak patterns and high intrastrain spectral similarity were consistently observed across technical replicates.

PCA, spectral stacking, and dendrogram analyses were performed using ClinProTools® version 3.0 (Bruker Daltonics), and the resulting MSPs were incorporated into an in-house *A. schubertii* database [27, 30]. Four closely related non-target species, *Aeromonas hydrophila*, *Aeromonas veronii*, *Aeromonas jandaei*, and *Aeromonas caviae*, were included as outgroup reference species for discriminatory and clustering analyses. These species were selected because of their phylogenetic relatedness to *A. schubertii* and their frequent association with freshwater fish diseases [27, 31].

### Validation method

The identification performance of the developed MALDI-TOF MS database was evaluated using 19 *A. schubertii* isolates, with particular emphasis on identification reliability and repeatability. Discriminatory performance was further assessed using the four closely related non-target *Aeromonas* species as outgroup controls.

MALDI-TOF MS identification scores were interpreted according to the manufacturer's recommendations. Scores <1.70 were classified as unreliable; scores of 1.70–1.99 indicated reliable genus-level identification; scores of 2.00–2.29 indicated reliable species-level identification; and scores ≥2.30 indicated highly reliable species-level identification. Repeatability was assessed by analyzing each isolate in triplicate and calculating the coefficient of variation (CV) of the corresponding log score values. A CV ≤10% was considered indicative of acceptable repeatability. Discriminatory performance was assessed by comparing protein spectral profiles and evaluating clustering patterns between *A. schubertii* and the four non-target *Aeromonas* species.

### Statistical analysis

Log score values were summarized as mean ± standard deviation from triplicate technical replicates. Repeatability was evaluated using the CV of log score values, with values ≤10% considered indicative of acceptable repeatability. Pearson correlation coefficients were calculated between corresponding processed spectral-intensity profiles of technical replicates to evaluate spectral reproducibility. PCA, spectral stacking, and dendrogram analyses were performed using ClinProTools® version 3.0 (Bruker Daltonics).

## RESULTS

MALDI-TOF MS identification performance: commercial versus custom MSP database

The identification performance of MALDI-TOF MS before and after incorporation of the custom MSP database is summarized in Table 2. Using the commercial Bruker database alone, 15/19 (78.95%) *Aeromonas schubertii* isolates achieved reliable species-level identification, with log scores ranging from 2.01 to 2.22. The remaining 4/19 (21.05%) isolates were identified only at the genus level, with log scores ranging from 1.95 to 1.98. These findings indicated incomplete species-level resolution of *A. schubertii* using the commercial reference database.

After incorporating the custom MSP database, all 19 isolates (100%) were correctly identified as *A. schubertii* at the species level, with highly reliable log scores ranging from 2.49 to 2.68. Thus, species-level identification increased from 78.95% with the commercial database to 100% with the custom MSP database, accompanied by higher identification confidence across all tested isolates. These findings demonstrate the diagnostic value of targeted enrichment of reference spectra for improving MALDI-TOF MS identification of *A. schubertii*.

The six representative *A. schubertii* isolates used for MSP construction exhibited distinct and reproducible protein mass spectral profiles. Although spectra were acquired across a mass range of 2–20 kDa, the characteristic profiles presented in Figure 1 were visualized predominantly within the 2–16 kDa range. Each isolate exhibited characteristic peak patterns, demonstrating both intraspecies spectral consistency and detectable interstrain variation.

Technical replicate spectra showed high reproducibility, with Pearson correlation coefficients ranging from 0.95 to 0.97. These findings indicated a high degree of similarity among replicate protein fingerprint profiles and supported the reproducibility of the MSPs generated from the six representative isolates.

**Table 2:** Comparative identification of A. schubertii isolates by Matrix-assisted laser desorption/ionization time-of-flight mass spectrometry using the commercial Bruker database and the custom MSP database.

**Isolate**	**Commercial Bruker MSP database**				**Custom MSP database**		
	**Identification**	**Log score**	**SD**		**Identification**	**Log score**	**SD**
CUVETASC01	*Aeromonas *sp.	1.95	0.049		*A. schubertii*	2.53	0.001
CUVETASC02	*A. schubertii*	2.01	0.028		*A. schubertii*	2.49	0.002
CUVETASC03	*Aeromonas *sp.	1.95	0.014		*A. schubertii*	2.63	0.001
CUVETASC04	*A. schubertii*	2.04	0.054		*A. schubertii*	2.68	0.003
CUVETASC05	*A. schubertii*	2.20	0.028		*A. schubertii*	2.64	0.00003
CUVETASC06	*Aeromonas *sp.	1.98	0.053		*A. schubertii*	2.66	0.0003
CUVETASC07	*A. schubertii*	2.09	0.036		*A. schubertii*	2.57	0.004
CUVETASC08	*A. schubertii*	2.06	0.038		*A. schubertii*	2.53	0.003
CUVETASC09	*Aeromonas *sp.	1.95	0.043		*A. schubertii*	2.54	0.028
CUVETASC10	*A. schubertii*	2.15	0.008		*A. schubertii*	2.61	0.019
CUVETASC11	*A. schubertii*	2.06	0.022		*A. schubertii*	2.64	0.003
CUVETASC12	*A. schubertii*	2.09	0.0002		*A. schubertii*	2.63	0.001
CUVETASC13	*A. schubertii*	2.01	0.068		*A. schubertii*	2.65	0.005
CUVETASC14	*A. schubertii*	2.15	0.0007		*A. schubertii*	2.62	0.003
CUVETASC15	*A. schubertii*	2.06	0.017		*A. schubertii*	2.67	0.001
CUVETASC16	*A. schubertii*	2.18	0.0001		*A. schubertii*	2.64	0.001
CUVETASC17	*A. schubertii*	2.20	0.007		*A. schubertii*	2.64	0.001
CUVETASC18	*A. schubertii*	2.22	0.003		*A. schubertii*	2.63	0.0001
CUVETASC19	*A. schubertii*	2.22	0.006		*A. schubertii*	2.64	0.001

MALDI-TOF MS = matrix-assisted laser desorption/ionization time-of-flight mass spectrometry; MSP = main spectra profile; SD = standard deviation. MALDI-TOF MS identification scores were interpreted as follows: <1.70 = unreliable identification; 1.70–1.99 = reliable genus-level identification; 2.00–2.29 = reliable species-level identification; and ≥2.30 = highly reliable species-level identification. Log score values are presented as mean values from triplicate technical replicates, with the corresponding SD for spectral characteristics and reproducibility.

**Figure 1:**
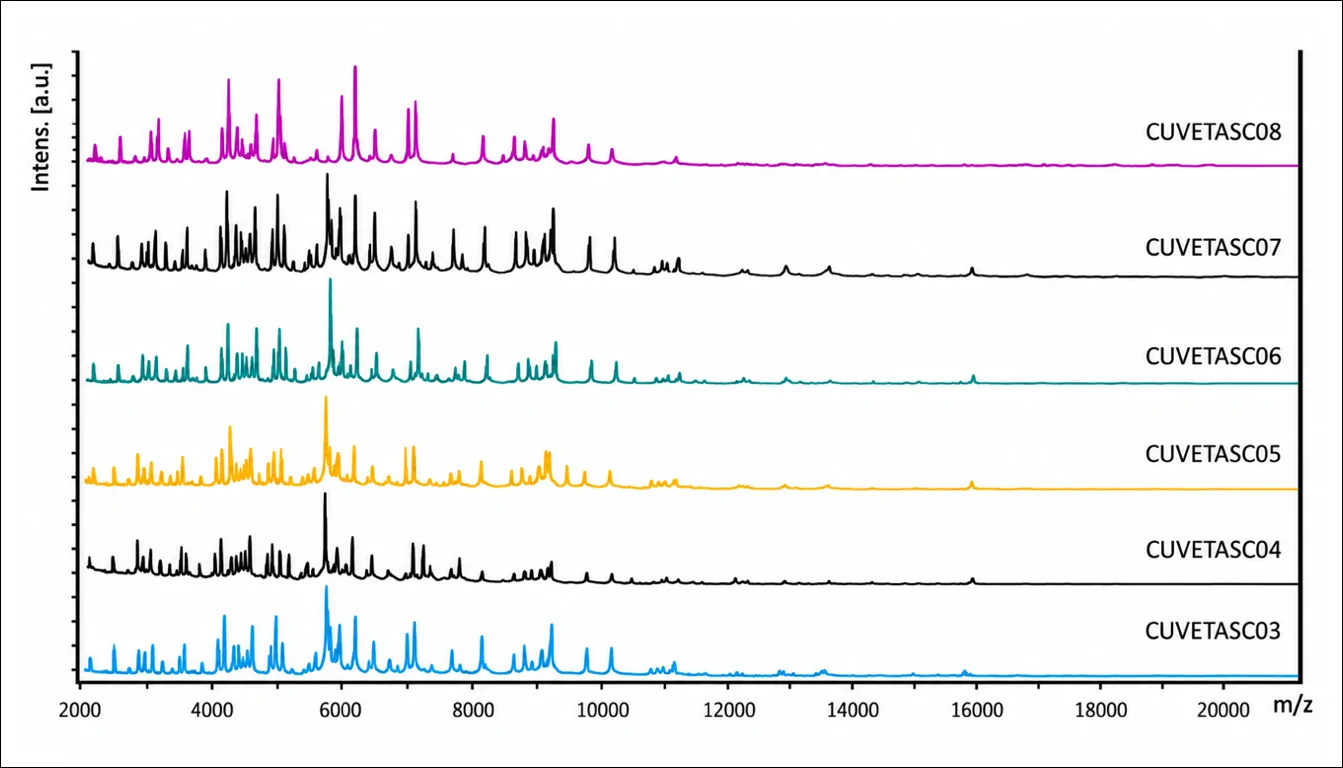
MALDI-TOF MS profiles highlighting characteristic peaks of six *A. schubertii* isolates. Spectra were acquired over a mass range of 2–20 kDa; the displayed profiles are shown within the 2–16 kDa range. MALDI-TOF MS = matrix-assisted laser desorption/ionization time-of-flight mass spectrometry; kDa = kilodalton.

### Spectral discrimination among *Aeromonas* species

In addition to the six *A. schubertii* isolates, four closely related non-target species, *Aeromonas hydrophila*, *Aeromonas veronii*, *Aeromonas jandaei*, and *Aeromonas caviae*, were included as outgroup controls. All isolates generated analyzable MALDI-TOF MS spectra following spectral preprocessing, including baseline correction, smoothing, normalization, peak detection, and outlier removal. Spectral stacking demonstrated distinct protein mass spectral patterns among *A. schubertii* and the non-target *Aeromonas* species (Figure 2).

**Figure 2:**
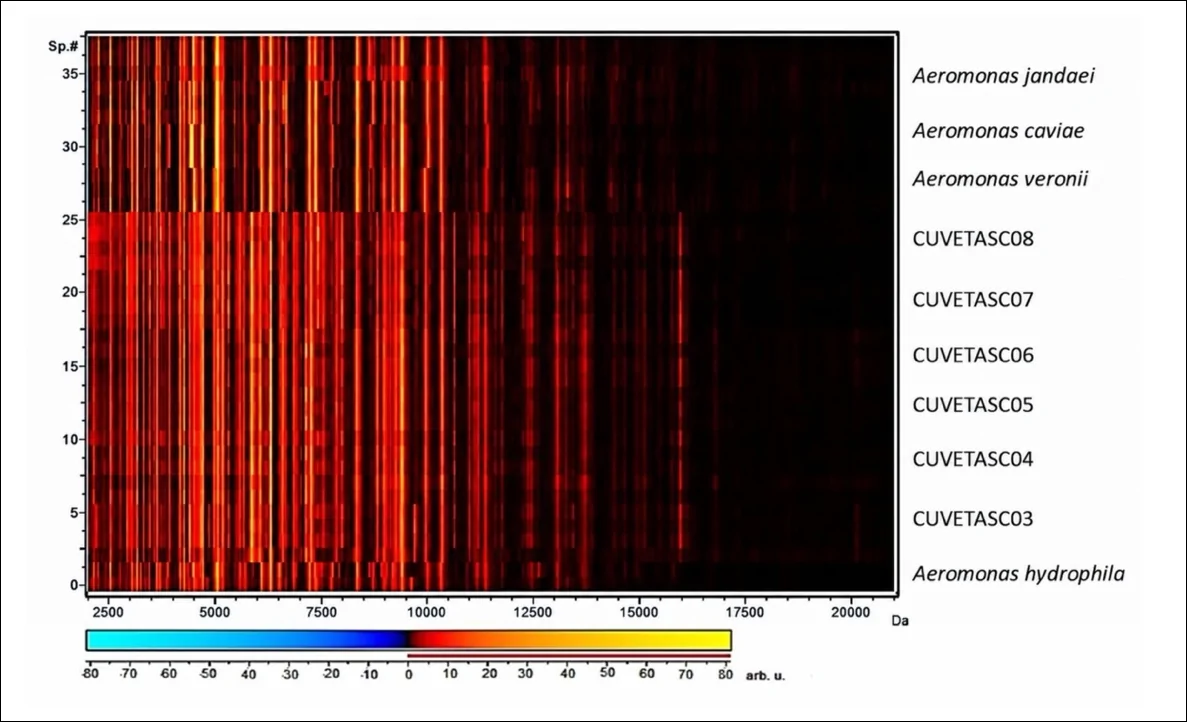
Gel stack view of MALDI-TOF MS spectra of *A. schubertii* and representative *Aeromonas* species. MALDI-TOF MS = matrix-assisted laser desorption/ionization time-of-flight mass spectrometry. The non-target reference species included *Aeromonas hydrophila*, *Aeromonas veronii*, *Aeromonas jandaei*, and *Aeromonas caviae*.

### Principal component analysis (PCA)

The *A. schubertii* isolates CUVETASC03–08 showed a distinct and relatively broad distribution in PCA space, particularly along PC1 and PC2 (Figure 3), indicating considerable intra-species variation among the isolates. Their separation from the reference *Aeromonas* species suggests that CUVETASC03–08 possess a distinct overall multivariate profile.

**Figure 3:**
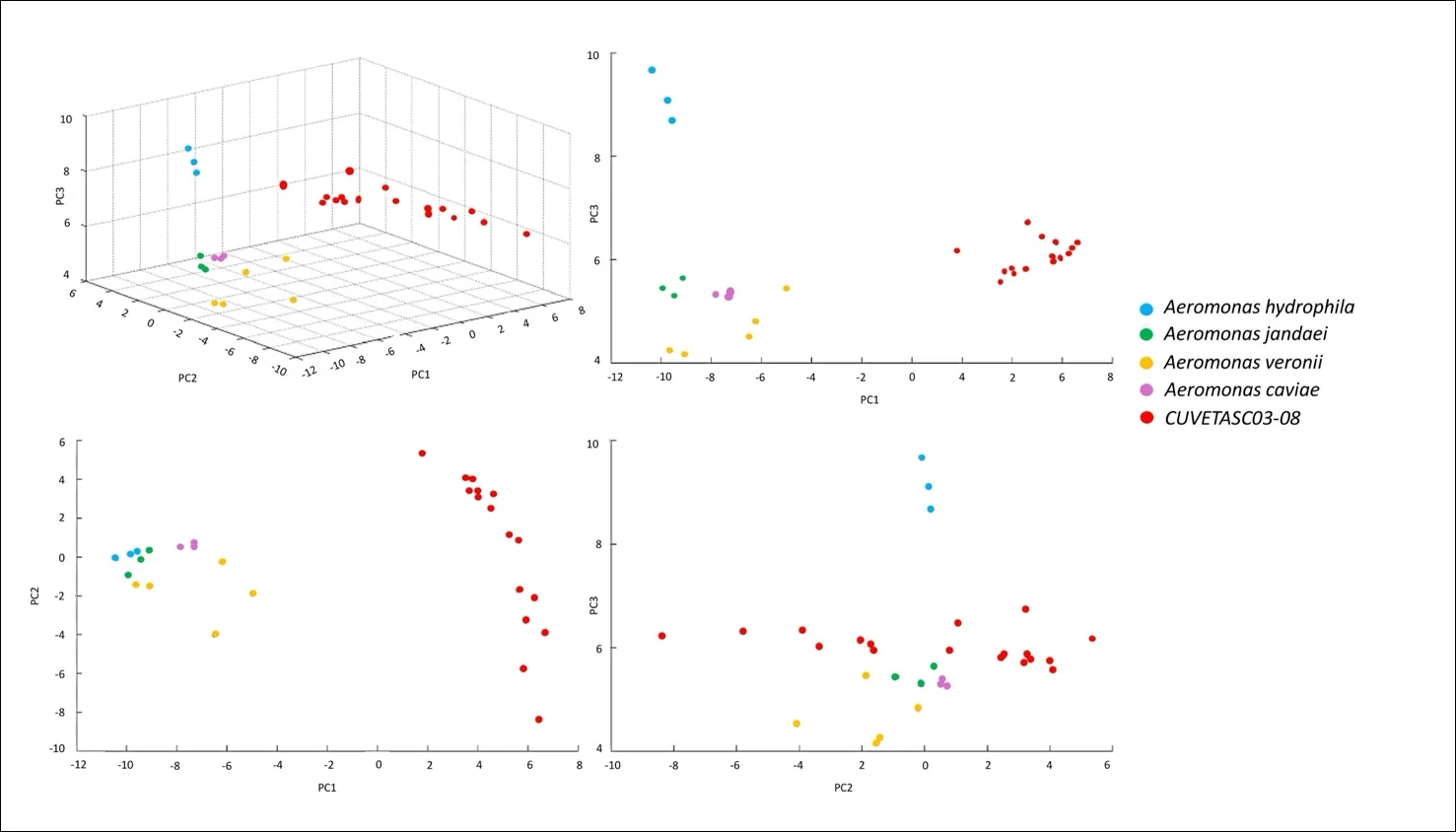
Principal component analysis (PCA) of *A. schubertii* from this study and other *Aeromonas* sp. isolates. The isolates, clustered in red colored dots, are *A. schubertii* used in this study. The other-coloured dots represent out-group *Aeromonas* sp. isolates.

### Dendrogram analysis

Dendrogram analysis further demonstrated clear differentiation between *A. schubertii* and the non-target Aeromonas species. The A. schubertii isolates clustered tightly within a distinct group, whereas *A. hydrophila*, *A. veronii,* *A. jandaei*, and *A. caviae* were separated from the *A. schubertii* cluster. The two major clusters merged at a linkage distance of approximately 0.75, whereas most within-cluster distances were <0.40, indicating comparatively high intraspecies spectral similarity and greater interspecies spectral divergence (Figure 4).

The clear separation of the four non-target *Aeromonas* species from *A. schubertii* in both PCA and dendrogram analyses supported the discriminatory capability of the custom MSP database.

**Figure 4:**
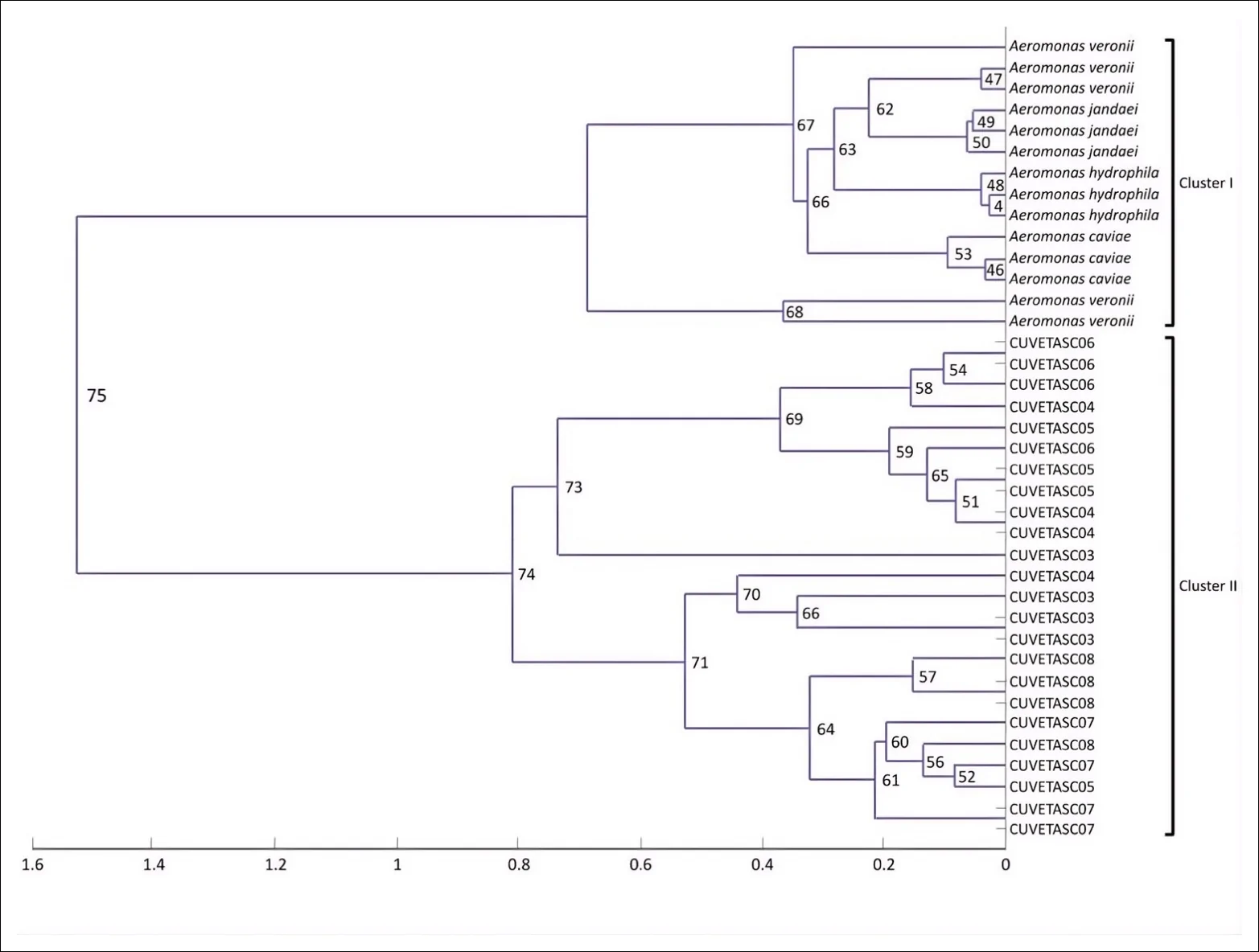
MSP dendrogram generated from MALDI-TOF MS spectra of *A. schubertii* and representative *Aeromonas* species. MSP = main spectral profile; MALDI-TOF MS = matrix-assisted laser desorption/ionization time-of-flight mass spectrometry. Non-target reference species comprised *A. hydrophila*, *A. veronii*, *A. jandaei*, and *A. caviae*.

### PCR assay, DNA sequencing, and phylogenetic analysis

PCR amplification of the *16S rRNA* and *gyrB* genes was successfully performed for all six isolates selected for MSP construction (CUVETASC03–CUVETASC08). Clear single amplicons corresponding to the expected sizes of approximately 1500 bp for the *16S rRNA* gene and 1100 bp for *gyrB* were observed, consistent with the positive controls (Figure 5). Subsequent Sanger sequencing followed by BLASTn analysis against the NCBI nucleotide database demonstrated high sequence similarity with reference *A. schubertii* sequences.

Sequence analysis demonstrated 99.68%–99.80% identity with reference *A. schubertii* strains across the evaluated 16S rRNA and *gyrB* sequences (Table 3). These findings supported the species-level taxonomic assignment of the six isolates used for MSP construction. Maximum-likelihood phylogenetic analyses were subsequently performed using approximately 1,500 bp of the *16S rRNA* gene and 1100 bp of *gyrB*.

The maximum-likelihood phylogenetic trees incorporated reference *A. schubertii* sequences retrieved from GenBank together with selected non-*A. schubertii* taxa. In both the *16S rRNA*- and *gyrB*-based trees, isolates CUVETASC03–CUVETASC08 clustered with established *A. schubertii* reference strains and formed a distinct clade, further supporting their taxonomic identification as *A. schubertii *(Figure 6).

**Figure 5:**
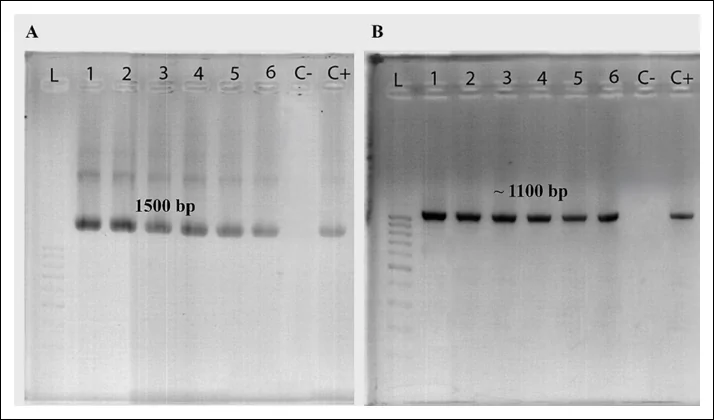
PCR products of the 16S rRNA (A) and *gyrB* (B) genes of *A. schubertii* separated on a 1.5% agarose gel. Lanes 1–6 correspond to isolates CUVETASC03–CUVETASC08; C− and C+ denote the negative and positive controls, respectively. PCR = polymerase chain reaction; C− = negative control; C+ = positive control; 16S rRNA = 16S ribosomal RNA; *gyrB* = DNA gyrase subunit B gene.

**Table 3:** Sequence-based analysis of A. schubertii isolates using 16S rRNA and gyrB sequences.

**Isolate**	***16S rRNA*** ** most closely related species/strain**	**Query coverage (%)**	**Identity (%)**	**Accession No.**	***gyrB*** ** most closely related species/strain**	**Query coverage (%)**	**Identity (%)**	**Accession No.**
CUVETASC03	*A. schubertii* WL-1	100	99.71	JQ303345.1	*A. schubertii* HYL1	100	99.80	HQ731454.1
CUVETASC04	*A. schubertii* LF1708	100	99.70	MG593845.1	*A. schubertii* HYL1	100	99.75	HQ731454.1
CUVETASC05	*A. schubertii* LF1708	100	99.70	MG593845.1	*A. schubertii* WL-4	100	99.75	MK416159.1
CUVETASC06	*A. schubertii* LF1708	100	99.68	MG593845.1	*A. schubertii* HYL1	100	99.80	HQ731454.1
CUVETASC07	*A. schubertii* LF1708	100	99.70	MG593845.1	*A. schubertii* WL-4	100	99.80	MK416159.1
CUVETASC08	*A. schubertii* LF1708	100	99.70	MG593845.1	*A. schubertii* WL-4	100	99.75	MK416159.1

16S rRNA = 16S ribosomal RNA; *gyrB* = DNA gyrase subunit B gene; No. = number. Query coverage represents the proportion of the query sequence aligned with the corresponding reference sequence in the database. Identity indicates the percentage of identical nucleotides between the query and reference sequences. Accession numbers refer to the corresponding GenBank reference sequences.

## DISCUSSION

The present study developed a species-specific MALDI-TOF MS MSP database for *A. schubertii* and demonstrated its potential to improve species-level identification of this emerging aquatic pathogen. The custom database addressed limitations observed with the commercial reference database, increasing species-level identification from 78.95% to 100% and producing highly reliable identification scores for all tested isolates. In addition, PCA and dendrogram analyses demonstrated clear spectral differentiation of *A. schubertii* from the evaluated fish-associated *Aeromonas* species. Given the increasing importance of *A. schubertii* in Southeast Asian aquaculture and its reported zoonotic potential, these findings support the value of species-specific spectral enrichment for improving the diagnosis and surveillance of an underrepresented aquatic bacterial pathogen.

### MALDI-TOF MS profiling and species differentiation of *Aeromonas schubertii*

The application of MALDI-TOF MS enabled characterization of *A. schubertii* isolates based on their protein mass spectral profiles. The generated spectra were highly reproducible among technical replicates, with Pearson correlation coefficients of 0.95–0.97, indicating consistent protein fingerprint profiles. Although gel stack visualization showed similarities among members of the genus *Aeromonas*, multivariate analyses provided greater discriminatory resolution. PCA demonstrated that *A. schubertii* isolates formed a distinct cluster separated from the evaluated non-target *Aeromonas* species, whereas dendrogram analysis similarly showed tight clustering of *A. schubertii* and separation from *Aeromonas hydrophila*, *Aeromonas veronii*, *Aeromonas jandaei*, and *Aeromonas caviae*.

The observed differentiation is consistent with previous findings indicating that *Aeromonas* species can exhibit characteristic protein mass fingerprints that facilitate species-level discrimination by MALDI-TOF MS [29]. The clustering pattern observed for *A. schubertii* is also consistent with its placement within *Aeromonas* group 501 reported by Benagli *et al.* [32], a group characterized by distinct phenotypic and genotypic features [33]. Collectively, the present spectral and clustering results support the capacity of MALDI-TOF MS to discriminate *A. schubertii* from the closely related species included in this study when appropriate reference spectra are available.

### Limitations of commercial MALDI-TOF MS spectral databases

Despite the advantages of MALDI-TOF MS, the commercial Bruker reference database provided incomplete species-level resolution for *A. schubertii*. Of the 19 isolates examined, 15 (78.95%) were reliably identified at the species level, whereas four (21.05%) achieved only genus-level identification. This finding illustrates an important limitation of reference database-dependent identification: diagnostic performance is influenced by the representation and quality of spectra available for the target organism. Underrepresentation of environmental and aquatic bacterial species in reference libraries may consequently reduce identification confidence for less frequently encountered pathogens such as *A. schubertii*.

**Figure 6:**
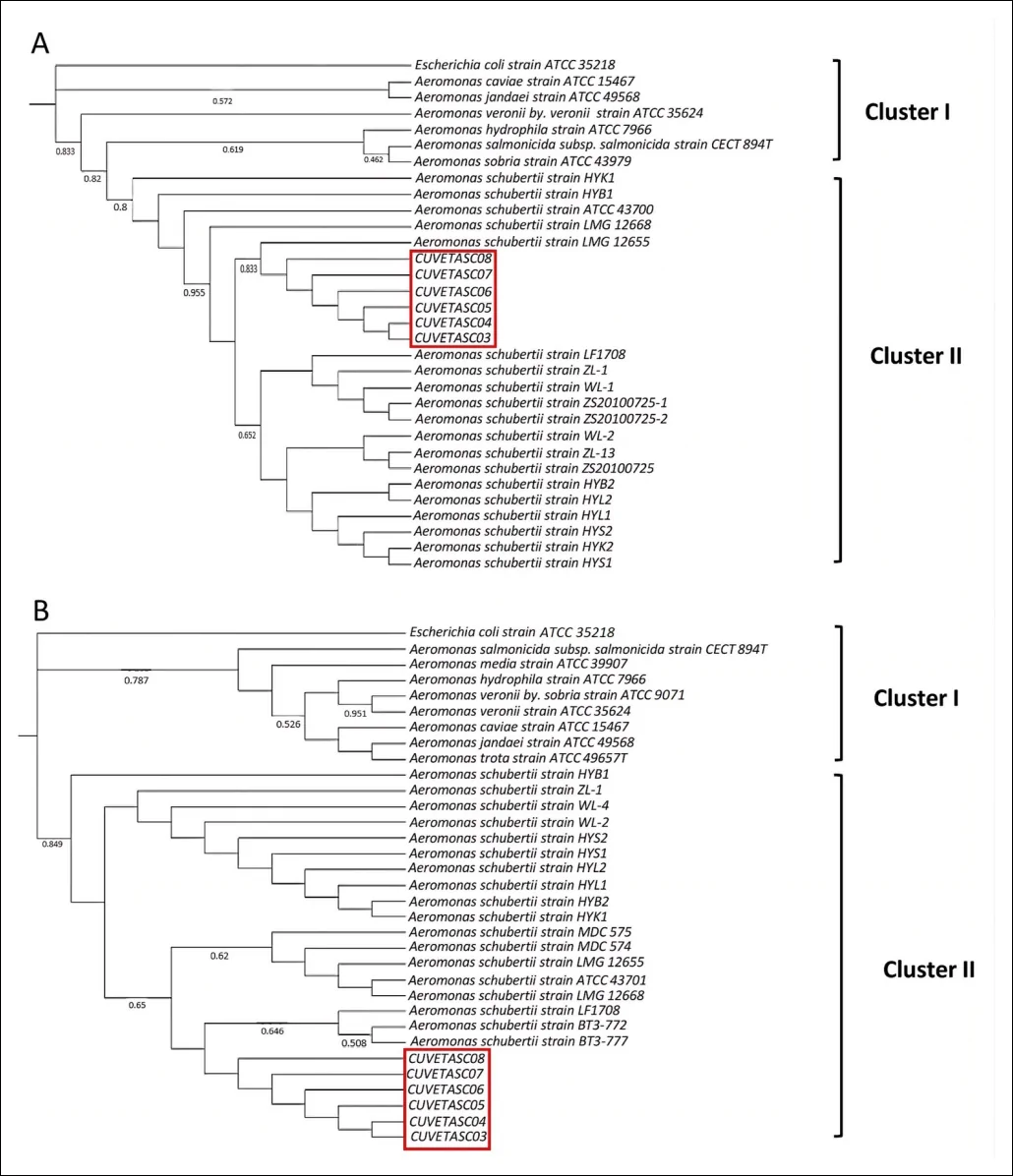
Maximum-likelihood phylogenetic analysis of *A. schubertii* and related bacteria based on 16S rRNA (A) and *gyrB* (B) sequences. Cluster I comprises the outgroup *Aeromonas* spp. and *Escherichia coli* ATCC 35218, whereas Cluster II comprises *A. schubertii*. Study isolates are indicated by black rectangles, and bootstrap values are shown at the branch nodes. *16S rRNA* = 16S ribosomal RNA; *gyrB* = DNA gyrase subunit B gene; ATCC = American Type Culture Collection. Bootstrap support was assessed using 1,000 replicates.

Identification performance may also be affected by methodological differences in sample preparation. Variations in extraction solvents and acid concentrations can influence protein recovery and spectral quality, thereby affecting the resulting identification profiles [34–36]. Organic acid-based extraction procedures preferentially recover abundant low-molecular-weight proteins, including ribosomal and nucleic acid-binding proteins; therefore, differences in extraction chemistry can alter the detectable protein profile [37]. Standardization of both sample preparation procedures and reference spectral libraries is consequently important when MALDI-TOF MS is applied to less well-represented aquatic bacterial species.

### Improvement of identification accuracy through a customized MSP database

Development of the custom *A. schubertii* MSP database substantially improved identification performance. Following incorporation of species-specific reference spectra, all 19 isolates (100%) were correctly identified at the species level, with log scores ranging from 2.49 to 2.68. In comparison, the commercial database yielded species-level identification for only 78.95% of the isolates, with log scores of 2.01–2.22 among those identified at the species level. Thus, targeted addition of high-quality *A. schubertii* reference spectra improved both the proportion of isolates identified at the species level and their associated identification confidence.

The custom database was evaluated using 19 *A. schubertii* isolates recovered from multiple freshwater fish hosts, including snakehead fish, Asian seabass, and hybrid red tilapia. This host representation provided an opportunity to evaluate the database against isolates originating from different fish hosts. However, the MSP library itself was constructed from six isolates obtained from diseased snakehead fish in Nakhon Pathom Province, Thailand. Therefore, the present findings should not be interpreted as demonstrating that the MSP library captures the full host-associated or geographic diversity of *A. schubertii*. Broader validation using isolates from additional hosts and geographic regions remains necessary.

The improvement achieved after database customization is consistent with previous studies demonstrating that MALDI-TOF MS identification of *Aeromonas* spp. is strongly dependent on reference database completeness [20, 29]. Difficulties in discriminating fish-associated *Aeromonas* species using commercial databases have previously been reported [29], whereas customized MSP libraries containing additional reference spectra have improved identification performance [20]. The increase in species-level identification from 78.95% to 100% observed in the present study further supports targeted spectral enrichment as a practical strategy for improving MALDI-TOF MS identification of underrepresented aquatic bacterial pathogens.

The capacity to expand MALDI-TOF MS databases also offers opportunities to incorporate spectra representing broader host, geographic, and strain diversity, potentially increasing their utility for epidemiological surveillance and outbreak investigations [38, 39]. Nevertheless, these potential applications require validation using appropriately diverse isolate collections before broader epidemiological utility can be established.

### Molecular validation using *16S rRNA* and *gyrB* gene analyses

Molecular analyses provided independent support for the taxonomic identification of the six isolates used for MSP construction. Sequence analysis of the *16S rRNA* and *gyrB* genes demonstrated 99.68%–99.80% identity with reference *A. schubertii* strains, and maximum-likelihood phylogenetic analyses showed that all six isolates clustered with established *A. schubertii* reference strains. These findings supported the species-level assignment of the isolates used to generate the custom MSP database.

Although *16S rRNA* gene sequencing is widely used for bacterial identification and phylogenetic characterization, its discriminatory capacity may be limited among closely related *Aeromonas* species, including *A. veronii*, *Aeromonas ichthiosmia*, and *Aeromonas simiae*. In contrast, housekeeping genes such as *gyrB* can provide greater discriminatory resolution because of their higher sequence variability while retaining conserved functional roles [40]. Furthermore, multilocus sequence analysis based on concatenated housekeeping genes has been reported to provide improved taxonomic and phylogenetic resolution within the genus *Aeromonas* [41–43]. The combined use of *16S rRNA* and *gyrB* analyses in the present study therefore provided complementary molecular evidence supporting the taxonomic identity of the isolates used for MSP construction.

### Implications for aquaculture diagnostics and pathogen surveillance

The findings indicate that MALDI-TOF MS, when supported by an appropriately curated species-specific MSP database, can provide rapid and reliable identification of *A. schubertii* following bacterial isolation. This capability may be particularly relevant to surveillance of white nodular disease, which has been associated with *A. schubertii* infection in freshwater fish. Because *A. schubertii* has also been reported in other aquatic hosts and opportunistic human infections, improved species-level identification may have relevance beyond freshwater fish diagnostics.

Combining MALDI-TOF MS protein fingerprinting with molecular confirmation also provides complementary approaches to bacterial characterization. Molecular methods can support taxonomic confirmation, whereas MALDI-TOF MS can facilitate rapid processing of cultured bacterial isolates once an appropriate reference database has been established. This combination may therefore strengthen laboratory identification and epidemiological surveillance, particularly for aquatic pathogens that are poorly represented in commercial spectral databases.

The customized MALDI-TOF MS approach may also offer practical workflow advantages compared with sequencing-based identification because MALDI-TOF MS enables rapid analysis of cultured colonies and supports high-throughput processing. However, this study did not formally compare turnaround time, workload, and cost. Consequently, the current data cannot quantify the relative economic and operational advantages of the custom MALDI-TOF MS approach over molecular identification methods. Future studies should directly compare diagnostic turnaround time, laboratory workload, and cost-effectiveness between these approaches.

Beyond its immediate application to *A. schubertii*, the developed MSP database may provide a foundation for expansion of reference spectra for aquatic *Aeromonas* species. Continued addition of well-characterized isolates could support the development of a broader aquatic *Aeromonas* MSP reference network. Future technological advances may also permit integration of such databases with portable or field-oriented MALDI-TOF MS platforms; however, the feasibility and diagnostic performance of such applications require dedicated validation.

### Limitations and future perspectives

Several limitations of the present study should be considered when interpreting the findings. First, the MSP database was constructed using only six *A. schubertii* isolates, all originating from diseased snakehead fish collected in Nakhon Pathom Province, Thailand. Although the database was subsequently evaluated using 19 isolates originating from Thailand and Vietnam and from multiple fish hosts, this collection may not adequately represent the broader genetic and proteomic diversity of *A. schubertii* across different geographic regions, hosts, and production systems. Second, MALDI-TOF MS performance can be influenced by sample preparation procedures, instrument settings, calibration, and laboratory-specific workflows, which may affect the transferability and reproducibility of the database across laboratories.

Third, validation was performed within the study setting and did not include independent external or multicenter laboratory validation. Although the discriminatory analyses included four closely related non-target *Aeromonas* species, a broader panel of phylogenetically and diagnostically relevant bacterial species would provide a more comprehensive assessment of specificity. Fourth, whole-genome sequencing was not performed; therefore, direct comparison of MALDI-TOF MS identification with genome-based taxonomic approaches was beyond the scope of the study. Finally, formal analyses of diagnostic turnaround time and cost-effectiveness were not undertaken.

Future studies should expand the MSP database using a larger collection of molecularly and genomically characterized *A. schubertii* isolates representing diverse hosts, geographic regions, and epidemiological settings. Multicenter validation should assess interlaboratory reproducibility and database transferability across different MALDI-TOF MS workflows. Inclusion of a broader range of non-target *Aeromonas* species and other phenotypically similar aquatic bacteria would further strengthen assessment of diagnostic specificity. Integration with whole-genome sequencing and multilocus sequence approaches may also help characterize strain diversity and determine whether spectral variation corresponds to genetically distinct lineages. In addition, prospective diagnostic studies comparing MALDI-TOF MS with conventional and sequencing-based methods in terms of accuracy, turnaround time, workload, and cost would help establish its practical value for routine aquaculture diagnostics.

## CONCLUSION

This study developed and validated a species-specific MALDI-TOF MS MSP database for *A. schubertii*, addressing the limited representation of this emerging aquatic pathogen in the commercial reference database. The custom MSP database increased species-level identification from 78.95% with the commercial database to 100%, with all 19 tested isolates achieving highly reliable log scores of 2.49–2.68. High reproducibility of technical replicate spectra, with Pearson correlation coefficients of 0.95–0.97, further supported the consistency of the generated protein fingerprints. PCA and dendrogram analyses clearly differentiated *A. schubertii* from the four evaluated non-target *Aeromonas* species. Molecular characterization based on 16S rRNA and *gyrB* sequences, together with phylogenetic analyses, further supported the taxonomic identity of the isolates used to construct the MSP.

The developed MSP database provides a practical approach for improving MALDI-TOF MS-based identification of *A. schubertii* following bacterial isolation and may strengthen laboratory diagnosis and surveillance of white nodular disease in freshwater aquaculture. A major strength of the study was the integration of proteomic profiling with molecular confirmation and multivariate spectral analyses. In addition, evaluation of the database using 19 *A. schubertii* isolates from multiple fish hosts, and inclusion of four closely related non-target *Aeromonas* species provided evidence of identification performance and spectral discrimination within the evaluated isolate collection. Expanding MALDI-TOF MS reference libraries also provides a framework for improving identification of other aquatic bacterial pathogens underrepresented in commercial databases.

The species-specific MSP database substantially improved MALDI-TOF MS identification of *A. schubertii* within the evaluated isolate collection and provided clear spectral differentiation from the tested non-target *Aeromonas* species. These findings support targeted enrichment of MALDI-TOF MS reference databases as a promising strategy for improving the identification of underrepresented aquatic bacterial pathogens. With broader isolate representation and independent multicenter validation, the developed database could contribute to more rapid and reliable diagnosis and surveillance of *A. schubertii*-associated disease in freshwater aquaculture.

## DATA AVAILABILITY

The custom MSP database and associated MALDI-TOF MS spectral data are available from the corresponding authors upon reasonable request for research and validation purposes.

## GENERATIVE AI DECLARATION

During the preparation of this manuscript, the authors used generative artificial intelligence tools solely for language editing, grammar correction, and improvement of clarity and readability. The authors critically reviewed and revised all AI-assisted content and take full responsibility for the accuracy, integrity, and originality of the final manuscript. No generative AI tools were used to generate, analyze, or interpret the scientific data or results.

## AUTHORS’ CONTRIBUTIONS

MFM: Conceptualization, investigation, methodology, formal analysis, and writing – original draft. HB: Investigation. MM: Investigation and writing – review and editing. SFA: Investigation and writing – review and editing. RHS: Investigation and writing – review and editing. CR: Conceptualization, supervision, methodology validation, funding acquisition, and writing – review and editing. All authors have read and approved the final manuscript.
